# NIA Office of Special Populations

**DOI:** 10.1093/geroni/igab046.1046

**Published:** 2021-12-17

**Authors:** Patricia Jones

**Affiliations:** National Institute on Aging, Bethesda, Maryland, United States

## Abstract

Dr. Jones will discuss the work of the NIA Office of Special Populations. Dr. Jones will also be available for small group discussion.

